# Antagonist Effects of Veratric Acid against UVB-Induced Cell Damages

**DOI:** 10.3390/molecules18055405

**Published:** 2013-05-10

**Authors:** Seoung Woo Shin, Eunsun Jung, Seungbeom Kim, Kyung-Eun Lee, Jong-Kyung Youm, Deokhoon Park

**Affiliations:** 1 Biospectrum Life Science Institute, EinesPlatz 11th Fl., 442-13 Sangdaewon Dong, Seoungnam City, Gyunggi-do 462-807, Korea; 2 COSMAX R&I Center, PangyoInnoValleyE #306, 255, Pangyo-ro, Bundang-gu, Seongnam-si, Gyeonggi-do 463-400, Korea

**Keywords:** veratric acid, photoprotection, anti-inflammation

## Abstract

Ultraviolet (UV) radiation induces DNA damage, oxidative stress, and inflammatory processes in human epidermis, resulting in inflammation, photoaging, and photocarcinogenesis. Adequate protection of skin against the harmful effect of UV irradiation is essential. In recent years naturally occurring herbal compounds such as phenolic acids, flavonoids, and high molecular weight polyphenols have gained considerable attention as beneficial protective agents. The simple phenolic veratric acid (VA, 3,4-dimethoxybenzoic acid) is one of the major benzoic acid derivatives from vegetables and fruits and it also occurs naturally in medicinal mushrooms which have been reported to have anti-inflammatory and anti-oxidant activities. However, it has rarely been applied in skin care. This study, therefore, aimed to explore the possible roles of veratric acid in protection against UVB-induced damage in HaCaT cells. Results showed that veratric acid can attenuate cyclobutane pyrimidine dimers (CPDs) formation, glutathione (GSH) depletion and apoptosis induced by UVB. Furthermore, veratric acid had inhibitory effects on the UVB-induced release of the inflammatory mediators such as IL-6 and prostaglandin-E2. We also confirmed the safety and clinical efficacy of veratric acid on human skin. Overall, results demonstrated significant benefits of veratric acid on the protection of keratinocyte against UVB-induced injuries and suggested its potential use in skin photoprotection.

## 1. Introduction

Solar ultraviolet light can penetrate the atmosphere and cause most of the commonknown skin disorders [1]. Acute UV irradiation can elicit various responses including sunburn, inflammation, DNA damage, and apoptosis [2,3,4]. Chronic and repetitive UV irradiation can lead to photoaging, sustained immune suppression, and carcinogenesis of the skin [2,5].The UVB range of solor radiation can penetrate theskinepidermis, inducing both direct and indirect DNA damaging effects. UV radiation depletes the cutaneous defense system and leads to the accumulation of DNA damages, excessive cell apoptosis, skin aging and impairs the epidermal integrity [6,7].

**Figure 1:**
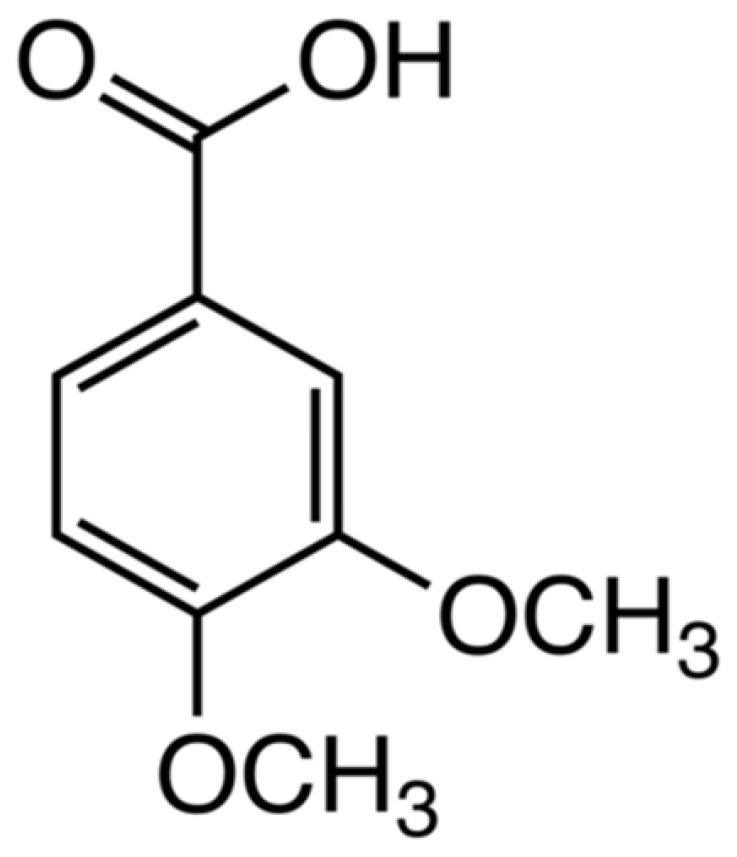
Chemical structure of veratric acid.

There has been a considerable interest in the use of naturally occurring botanicals for the protection of human skin from UV-induced damage as flavonoids and other phenolic compounds have been considered as a major class of protectants against UV-induced damage [8,9,10]. Recently, mushrooms have become attractive sources of bioactive compounds, including phenolic compounds that have antioxidative [11], antitumor [12], antimicrobial [13], and anti-inflammatory properties [14]. Among the various edible and medicinal mushrooms, *Sparassiscrispa* has been reported to contain a b-1,3-D-glucan that enhances antitumor responses [15]. In addition, *S. crispa* contains veratric acid (Figure 1), which has been detected in some Korean medicinal mushrooms [16]. It has been reported that veratric acid has antioxidative and anti-inflammatory properties. In hypertension-induced rats, veratric acid decreased the blood pressure by reducing the NO concentration and attenuating oxidative stress [17,18]. However, other possible novel functions of veratric acid in skin biology remain elusive. In this study we investigated the protective effects of veratric acid on UV-induced damage in keratinocytes. We also evaluated the skin recovery effects and safety after topical application of veratric acid on humans to determine the feasibility of clinical use.

## 2. Results and Discussion

### 2.1. Veratric Acid Inhibits UV-Induced Damage in HaCaT Cells

**Figure 2:**
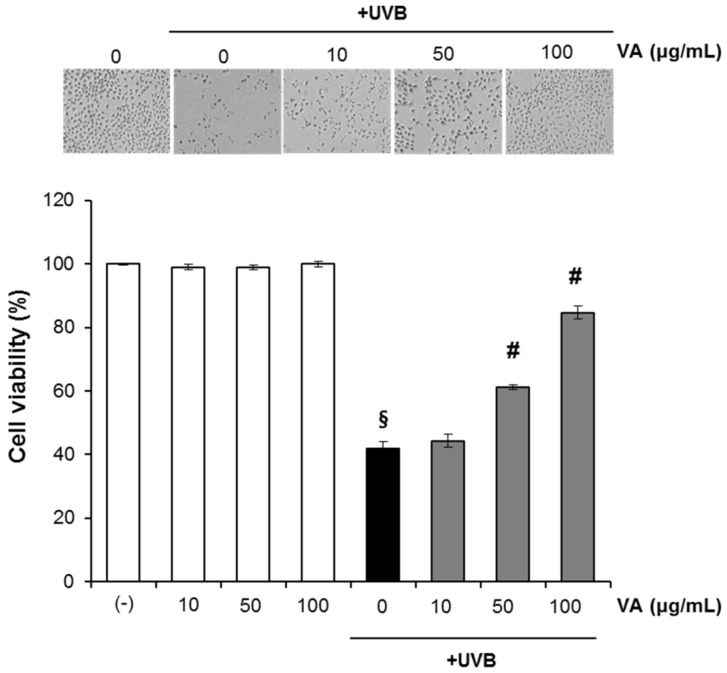
Veratric acid treatment protects HaCaT cells against UVB-mediated phototoxicity.

We initially investigated the effect of veratric acid (10–100 µg/mL) on the UVB-mediated decrease in cell viability. HaCaT cells were treated with different concentrations of veratric acid (10–100 μg/mL) for 12 h after being exposed to UVB. The percentage of viable cells was assessed by using the MTT assay at 12 h after UVB irradiation. As expected, UVB (20 mJ/cm^2^) irradiation of HaCaT cells resulted in decreased cell viability. This UVB-induced cell death was significantly attenuated by treatment of cells with veratric acid (10–100 µg/mL) in a concentration-dependent manner as determined by the MTT assays (Figure 2). Veratric acid alone (w/o UVB) did not show any significant effects on cell viability at the treated concentration(Figure 2).

*p* < 0.01 compared with the vehicle-treated group; # *p* < 0.01 compared with the UVB treated group; (N = 5); VA: veratric acid.

**Figure 3:**
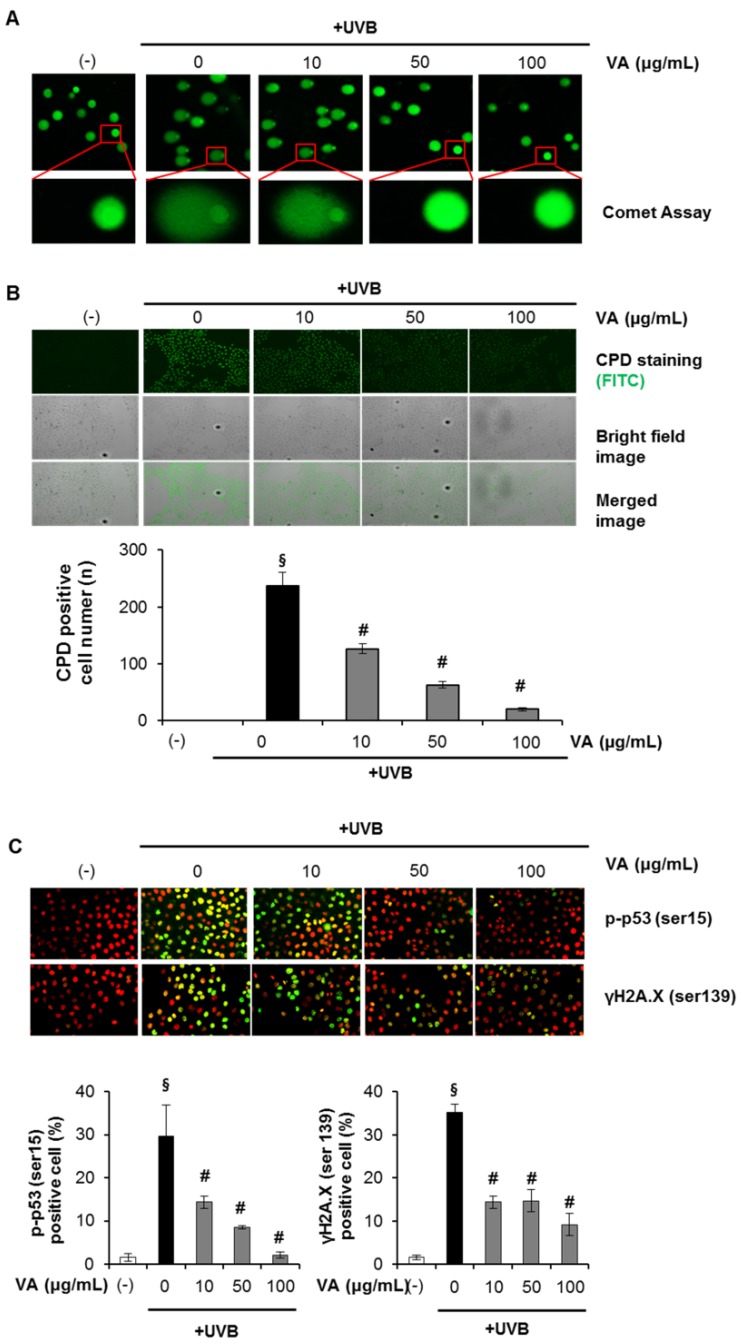
Effect of veratric acid on UVB-induced DNA damage. (**A**) Analysis of DNA damage by the comet assay. (**B**) Cyclobutane pyrimidine dimer (CPD) quantification. (**C**) Immunofluorescence analysis of phophoactive histone H2A.X (γH2A.X) and phospho-p53 (Serin15).

Then we determined and verified the photoprotective effect of veratric acid on UVB-induced cellular DNA damage. Cells were treated with different concentrations of veratric acid (10–100 μg/mL) for 12 h after being exposed to UVB (20 mJ/cm^2^). At 12 h after UVB irradiation, the cellular DNA damage was detected by comet assay, CPDs formation assay and analyzing phospho-p53 (Ser-15) and γ-H2AX expression levels. The DNA damaging effect and its prevention by veratric acid were determined by measuring comet tail length under a microscope. As shown in Figure 3A, exposure of HaCaT cells to UVB radiation (20 mJ/cm^2^) resulted in extensive DNA damage, as reflected by the comet tail length, compared to cells that were not exposed to UVB radiation. However, treating cells with veratric acid (10–100 µg/mL) resulted in a reduced amount of DNA damage compared to cells not treated with veratric acid, but exposed to UVB, as evidenced by the comet structure.

*p* < 0.01 compared with the vehicle-treated group;^#^ *p* < 0.01 compared with the UVB treated group; (N = 3); VA: veratric acid.

Then we have checked the effect of veratric acid on UVB-induced DNA damage in the form of CPDs formation. CPD represents major UVB-induced DNA damage, therefore, CPD formation was determined to evaluate the protective effect of veratric acid.The number of CPD-positive cells and intensity of staining of CPD-positive cells was markedly decreased in veratric acid-treated cells compared to the cells which were not treated with veratric acid, but exposed to UVB (Figure 3B).

The kinetics of UVB-induced DNA damage was also investigated by analyzing phospho-p53 (Ser-15) and γ-H2AX expression levels. UVB irradiation induced p53 (Ser-15) and γ-H2AX phosphorylation in a dose-dependent manner in HaCaT cells (data not shown). In addition, we determined the intracellular location of phospho-p53 (Ser-15) and γ-H2A.X proteins in HaCaT cells using immunofluorescence staining and confocal microscopy. When the cells were analyzed 12 h after UVB irradiation, the number of FITC positive cells (p-p53, γH2A.X) and intensity of staining of FITC-positive cells was markedly decreased in veratric acid-treated cells compared to the cells which were not treated with veratric acid but exposed to UVB (Figure 3C). Taken together, these results suggest that veratric acid reduced UVB-induced DNA damage compared to that in UVB alone-exposed control cells. HaCaT cells used in our study are spontaneously immortalized through mutations of p53 gene. Earlier studies with this cell line have argued for their appropriateness and as a closest model to normal keratinocytes [19], but the results obtained in cells with wildtype p53 may be very different (apoptosis, cell cycle regulation, *etc.*).

### 2.2. Veratric Acid Increases S-Phase Population in UVB-Irradiated Cells

**Figure 4:**
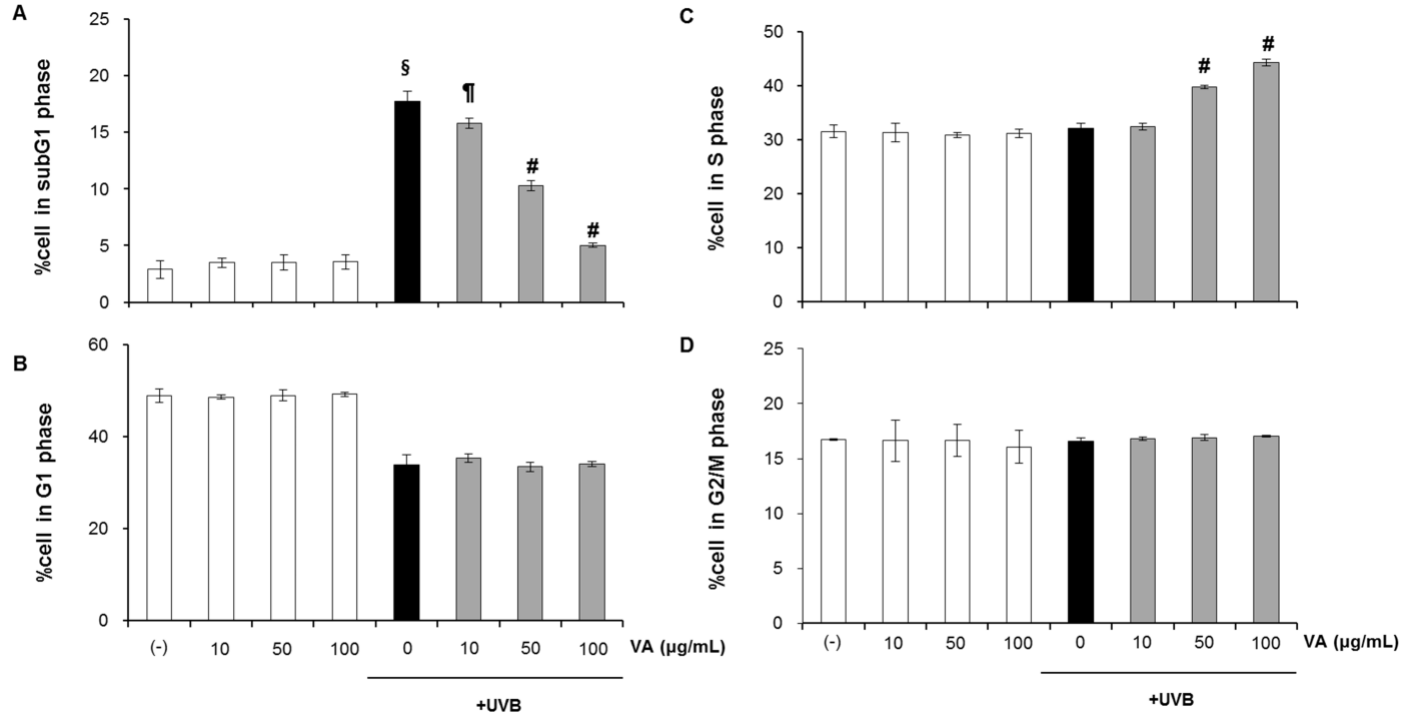
Accumulation of HaCaT cells in S phase following veratric acid treatment. The percentage of cells in the (**A**) subG1, (**B**) G1, (**C**) S, (**D**) G2/M phase.

In response to DNA damage, eukaryotic cells cease to progress through the cell cycle and arrest at specific checkpoints which serve to maintain genomic integrity. We, therefore, examined the effect of veratric acid in modulating cell cycle following UVB irradiation (Figure 4). HaCaT cells were treated with veratric acid (10–100 μg/mL) for 12 h after irradiation with 20 mJ/cm^2^ UVB. At 12 h after UVB irradiation, cells were trypsinized, stained with propidium iodide and analzed for DNA content using flow cytometry. Upon exposure to 20 mJ/cm^2^, the sub-G1 population was significantly increased to (16.69% versus 2.15% in control) (Figure 4A), largely at the expense of a decrease in G1 phase cells (36.24% versus 50.51% in control) (Figure 4B).

*p* < 0.01 compared with the vehicle-treated group;^#^ *p* < 0.01 compared with the UVB treated group; (N = 3); VA: veratric acid.

Post-treatment with veratric acid, however, reversed the UVB-caused sub-G1 increase, resulting in an increase in S phase cells (43.86% in UVB+Veratric acid 100 µg/mL versus 31.1% in UVB alone) (Figure 4A,B). These findings show that post-irradiation veratric acid treatment resulted in cessation in cell division and accumulation of UVB irradiated cells in S phase, suggesting that it allows more time for the cellular repair of DNA damage.

### 2.3. Veratric Acid Protects HaCaT Cells from UVB-Mediated Apoptosis

Apoptotic cells were estimated by calculating the number of subdiploid cells in the cell cycle histogram. HaCaT cells were treated with veratric acid (10–100 μg/mL) for 12 h after irradiation with 20 mJ/cm^2^ UVB. The percentage of cell population at sub-G1 phase as measured by flow cytometry.

**Figure 5:**
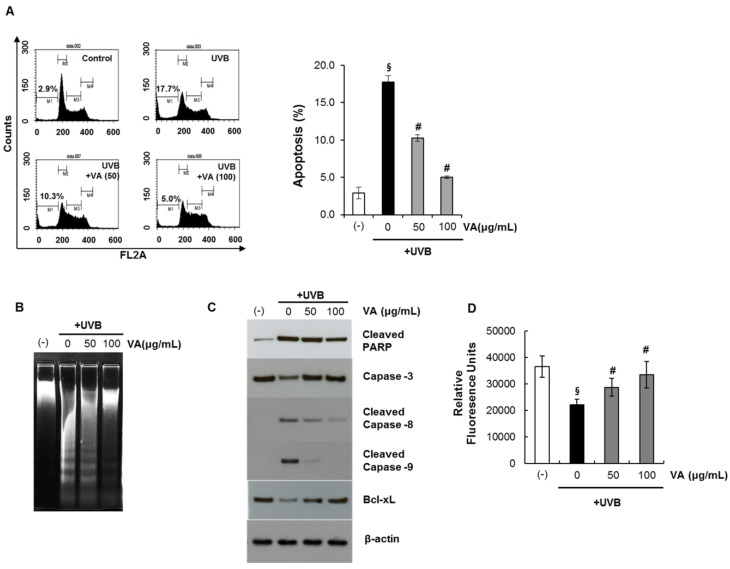
Inhibitory effect of veratric acid on UVB-induced apoptosis. (**A**) The percentage of apoptotic cells. (**B**) Agarose gel electrophoresis of nuclear DNA fragments. (**C**) Immunoblot analysis of various apoptosis related proteins. (**D**) Intracellular GSH level.

As shown in Figure 5A, a substantial increase in the number of apoptotic cells was observed on exposure to UVB. Veratric acid itself did not induce apoptosis at these concentrations. We found that treating HaCaT cells with veratric acid after UVB irradiation prevented UVB-mediated apoptosis. Next, UV-triggered apoptosis in HaCaT cells was determined by measuring DNA using agarose gel electrophoresis (Figure 5B).

*p* < 0.01 compared with the vehicle-treated group;^#^ *p* < 0.01 compared with the UVB treated group; (N = 3); VA: veratric acid.

DNA fragmentation decreased significantly in veratric acid-treated cells after exposure to UVB compared to that in untreated cells. We also evaluated changes in apoptotic marker proteins as a result of UVB irradiation and the influence of veratric acid on these proteins.Previous studies have identified caspases as important apoptosis mediators induced by a range of stimuli [20]. Activation of capase-3,-8 and -9 in HaCaT cells was assessed by immunoblot analysis of lysates from cells that had been exposed to UVB; with and without veratric acid treatment.Asexpected; we found that UVB irradiation resulted in activated caspase-3,-8 and 9 indicating thattheproform of caspase was cleaved into the active form. However; the cleavage was significantly reduced by veratric acid (Figure 5C). UVB irradiation also induced the formation of PARP proteolytic cleavage fragments; indicating imminent apoptosis. The cleaved PARP product markedly increased in untreated cells compared to that in veratric acid-treated cells upon exposure to UVB (Figure 5C). Taken together; UVB induced procaspase-3 cleavage into the active caspase-3 form; and caspase-3 induced PARP degradation. The results also indicate that veratric acid exhibited a protective effect on UVB-induced apoptosis.

### 2.4. Veratric Acid Prevent UVB-Induced Depletion of GSH in HaCaT Cells

Glutathione (GSH) depletion is a central signaling event that regulates the activation of cell death pathways. GSH depletion is often taken as a marker of oxidative stress and thus, as a consequence of its antioxidant properties scavenging ROS. GSH is well-known antioxidant which is usually present as the most abundant low-molecular-mass thiol in most organisms. It can act as the electron donor for glutathione peroxidase in animal cell, and also directly reacts with ROS [21]. Cells were treated with different concentrations of veratric acid after being exposed to UVB (20 mJ/cm^2^). After 12 h, cellular GSH levels were determined with the GSH-sensitive fluorescent dye MCB.The total GSH levels fluorescence units of MCBin HaCaT cells exposed to UVB were significantly decreased. However, the depletion of GSH was significantly protected by the treatment of veratric acid. (Figure 5D). These results indicate that veratric acid is able to regulate the cellular redox signaling events modulating cell death activation and progression by prevention of depleting GSH.

### 2.5. Veratric Acid Inhibits UVB-induced Inflammation *in Vitro* and *in Vivo*

**Figure 6:**
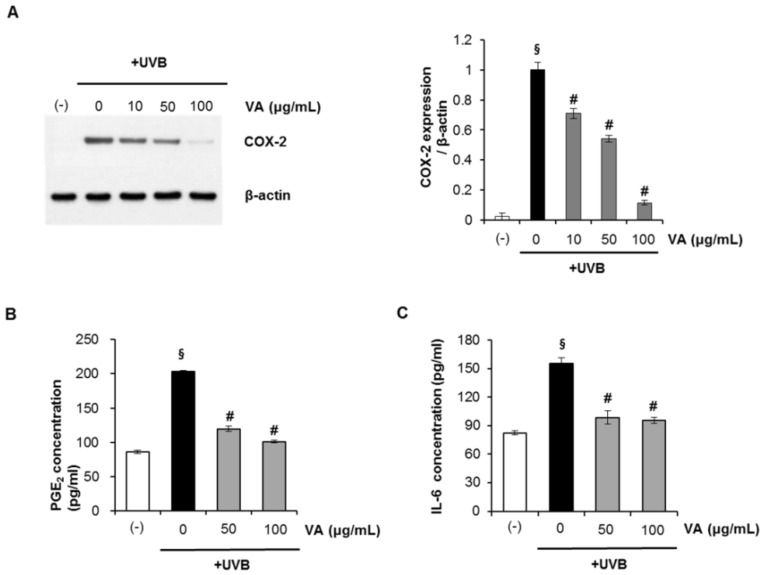
Veratric acid suppresses UVB-mediated inflammation cytokine production in human HaCaT keratinocytes. (**A**) Cyclooxygenase (COX)-2 protein expression. (**B**) Prostaglandin-E_2_ (PGE_2_) and (**C**) Interleukin-6 (IL-6) concentrations in cell culture superantants.

Abnormal upregulation of COX-2 and inflammation play an important role in skin cancer [22]. Studies have shown that UVB irradiation leads to MAPK activation [23] and triggers increased COX-2 expression, which catalyzes the formation of proinflammatory prostaglandins (e.g., PGE2) from arachidonic acid [24,25]. Here, we investigated whether veratric acid modulated COX-2 expression in UVB-irradiated HaCaT keratinocytes. HaCaT cells were treated with the veratric acid (10–100 μg/mL) after irradiation with 20 mJ/cm^2^ UVB. After 12 h, cyclooxygenase (COX)-2 protein expression was assessed by Western blotting. As shown in Figure 6A, UVB-induced COX-2 expression clearly decreased following treatment with veratric acid. The anti-inflammatory effects of veratric acid were further evaluated by analyzing UVB-induced production of pro-inflammatory cytokines such as PGE_2_ and IL-6. UVB (20 mJ/cm^2^) irradiation markedly upregulated PGE_2_ and IL-6, which were suppressed by treatment with veratric acid after UVB irradiation (Figure 6B,C). These results suggest that veratric acid has anti-inflammatory activity.

*p* < 0.01 compared with the vehicle-treated group; ^#^ *p*< 0.01 compared with the UVB treated group; (N = 4); VA: veratric acid.

**Figure 7:**
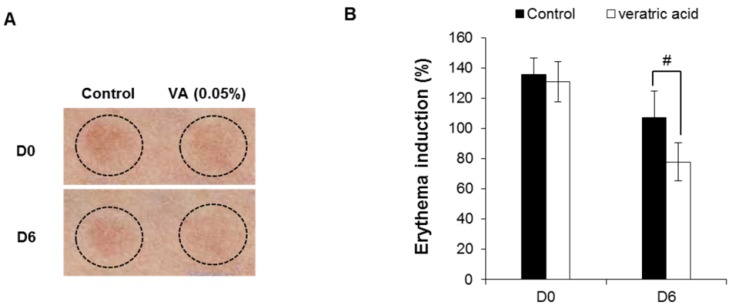
Skin recovery effect of veratric acid were evaluated in human studies. (**A**) Digital images. (**B**) Induction percentage of eythema.

^#^ *p* < 0.01 compared with the vehicle-treated group.

The skin recovery effect of veratric acid was also evaluated *in vivo* using the UV erythema test (Figure 7). UV erythema test was performed in healthy eighteen female subjects (average age: 41.8±4.1 years) with Fitzpatrick skin type I, II, III who had no history of allergenic contact dermatitis. The investigator fully explained the purpose and procedures of the study, schedule, compensation, and anticipated adverse reactions or side effects.

Test areas on the forearms of 18 healthy subjects were treated with veratricacidor the vehicle for 30 min. Subsequently, the test areas were irradiated with 1.5-fold the MED and the induced erythema was measured photometrically at the first visit (base line: before UV irradiation), 0 (after UV irradiation), 1, 2, 3 and 6 days after application of veratric acid*.* The erythema index was evaluated using Mexameter® MX18 (C+K, Cologne, Germany). Compared to control group, veratric acid group significantly reduced UV-induced skin erythema at 6 days after topical application (Figure 7A,B). Veratric acid showed skin recovery effect on UV-induced damaged skin in clinical study.

### 2.6. Human Skin Primary Irritation Test of Veratric Acid

To evaluate the irritation effect of veratric acid for clinical applications to human skin, a patch test was performed. Thirty healthy Korean subjects with Fitzpatrick skin type I, II, III were selected on the basis of inclusion and exclusion criteria, and written consent was obtained in each case. The average age was 38.1 years (range 24–47: all females). In our study, as shown in Table 1, none of the 30 subjects experienced a reaction based on the 48 and 72 h readings. Specifically, we did not observe any adverse reactions such as erythema, burning or pruritus in the study subjects that was related to the topical treatment of veratric acid.

**Table 1:** Human skin primary irritation test.

No.	Test material	48 h					72 h					Reaction grade ^b^		
		±	1+	2+	3+	4+	±	1+	2+	3+	4+	48 h	72 h	Mean
1	Squalene	− ^a^	−	−	−	−	−	−	−	−	−	0	0	0
2	VA (0.1%)	−	−	−	−	−	−	−	−	−	−	0	0	0

^a^ No reaction; ^b^ Reaction grade=∑ [{Grade × No. of Responders}/{4 (Maximum grade) × 30 (Total Subjects)}] × 100 × (1/2); VA: veratric acid.

## 3. Material and Methods

### 3.1. Chemicals and Antibodies

The electrophoresis reagents and protein assay kit were purchased from Invitrogen (Carlsbad, CA, USA). Antibodies against cleaved poly (ADP-ribose) polymerase (PARP), pro-caspase 3, cleaved-caspase 8, cleaved-caspase 9 and Bcl-xL were obtained from Cell Signaling Technology (Beverly, MA, USA). COX-2 and β-actin were purchased from Santa Cruz Biotechnology (Santa Cruz, CA, USA). Monochlorobimane (MCB) were purchased from Molecular Probes (Eugene, OR, USA). Propidium iodide (PI) was purchased from Sigma Chemical Co. (St. Louis, MO, USA). Veratric acid (purity: 99%) was acquired from Santa Cruz Biotechnology and stock solution of veratric acid was dissolved in DMSO and stored at −20 °C. All other reagents were of analytical grade and purchased from Sigma.

### 3.2. Cell Culture

The HaCaThuman keratinocyte cell line was purchased from CLS (Eppelheim, Germany). The HaCaT cells was cultured in Dulbecco’s modified Eagle’s medium (DMEM) supplemented with 10% fetal bovine serum, 100 units/mL penicillin and 100 µg/mL streptomycin sulfate. Cells were incubated in a humidified atmosphere of 5% CO_2_ and 95% air at 37 °C. 

### 3.3. UV Irradiation and Treatment

For UV irradiation experiments, the HaCaT keratinocytes were cultured on 60 mm culture dishes for 48 hr. Then, the cells were incubated with 1% serum medium for 12 h and exposed to UVB irradiation using LZC-UVB lamp (Luzchem, Ottawa, ON, Canada), which had an emission spectrum of 280–370 nm and a peak at 312 nm. The UV dose was measured with a UV light meter UV-340 (Lutron, Coopersburg, PA, USA). After irradiation, the cells were replenished with 1% serum medium including veratric acid and followed for up to 12 h.

### 3.4. Cell Viability Assay

The cytotoxicity of veratric acid after UVB irradiation was determined using 3-[4,5-dimethylthiazol-2-yl]-2,5-diphenyltetrazolium bromide (MTT) reduction to the corresponding blue formazan by viable cells. Cells were grown to ~80% confluence and maintained in 1% serum medium for 12 h prior to UV exposure. The level of blue formazan was measured spectophotometrically and used as an indirect index of cell density. Briefly, cells were exposed to MTT (1 mg/mL) for 3 h at 37°C. The medium was removed, and the cells were solubilized with dimethyl sulfoxide. After complete solubilization, the presence of blue formazan was evaluated spectrophotometrically by measuring absorbance at 540 nm (reference, 620 nm) with an enzyme-linked immunosorbent assay (ELISA) plate reader. Viability was expressed as a percentage of the control.

### 3.5. Analysis of DNA Damage by the Comet Assay

UVB-induced DNA damage on a per cell basis was determined using the comet assay, as described previously [25]. HaCaT cells were exposed to UVB (20 mJ/cm^2^) and harvested 12 h later for the comet assay. Briefly, the cells were harvested and re-suspended in ice cold PBS after UVB treatment. Approximately 1 × 10^4^ cells in 80 µL of 0.5% (w/v) low melting point agarose were pipetted onto a frosted glass slide coated with a thin layer of 1.0% (w/v) agarose, covered with a coverslip, and placed on ice for 10 min. The cover slip was removed and the slides were immersed in ice-cold lysis solution containing 2.5 M NaCl, 10 mMTris, 100 mM Na_2_-EDTA, and 1% (w/v) N-lauroyl-sarcosine, pH 10.0, and 1.0% Triton X-100 was added immediately before use. After 2 h at 4 °C, the slides were placed into a horizontal electrophoresis tank filled with buffer (0.3 M NaOH, 1 mM EDTA, pH 13) and subjected to electrophoresis for 30 min at 300 mA. The slides were transferred to neutralization solution (0.4 M Tris-HCl) for 3 × 5 min washes and stained with ethidium bromide for 5 min. The comets were examined and photographed using a fluorescence microscope. Slides were viewed using the 20× objective of an Evos fluorescent microscope equipped with epifluorescence optics (Advanced Microscopy Group, Bothell, WA, USA).

### 3.6. Cyclobutane Pyrimidine Dimer (CPD) Quantification

To visualize CPD, HaCaT keratinocytes were seeded on 96 well black plate. HaCaT cells were exposed UVB (20 mJ/cm^2^) and stained with monoclonal antibody against CPD and secondary goat anti-mouse-FITC antibody according to the manufacturer’s instructions. Plates were imaged on a GE IN Cell Analyzer 1000 (GE Healthcare, Piscataway, NJ, USA), and the images were analyzed with GE IN Cell Analyzer1000 Workstation software.

### 3.7. Immunofluorescence

The cells were fixed in formaldehyde12 h after irradiation (20 mJ/cm^2^ UVB)and stained with using primary antibody (p-p53 S15; γ-H2AX) and the secondary antibody conjugated with Alexa Fluor 488/PI nuclear counterstain solution. Plates were imaged on a GE IN Cell Analyzer 1000, and the images were analyzed with GE IN Cell Analyzer1000 Workstation software. The number of Alexa Fluor 488-positive cells/100 PI-positive cells was determined in two individual high-power fields per experiment by two independent assessors.

### 3.8. FACS Analysis

Both adherent and floating cells were collected, washed with ice-cold PBS, and fixed with 70% ice-cold ethanol overnight at 4°C 12 h following UVB irradiation and/or veratric acid treatment. Fixed cells were washed twice with PBS and treated with 100 µg/mL RNase for 30 min at 37 °C and then stained with 1 mg/mL PI in PBS containing 0.05% Nonidet-P40. The cells were then analyzed with a FACScan flow cytometer (Becton Dickinson, Franklin Lakes, NJ, USA). The percentages of cells in different cell cycle phases were evaluated from an analysis of DNA histograms. Cells with a sub-G_0_/G_1_ DNA (sub-G_1_) were considered apoptotic cells.

### 3.9. Western Blot Analysis

Cells were washed twice with cold PBS and lysed in 150 µl of sample buffer (100 mMTris–HCl, pH 6.8, 10% glycerol, 4% SDS, 1% bromophenol blue, and 10% β-mercaptoethanol). The proteins were resolved on a NuPAGENovex 10% Bis-Tris Gel (Invitrogen). Following electrophoretic transfer of the proteins onto nitrocellulose membranes, they were subsequently hybridized with primary antibody (1:1,000) followed by a horseradish peroxidase-conjugated secondary antibody (1:2000). Finally, the protein bands were visualized using the PowerOpti-ECL Western Blotting Detection reagent (Anigen, Hwaseong, Korea). Protein bands were quantified with Image J software [26].

### 3.10. Intracellular GSH Level

Intracellular GSH levels were determined by using a GSH-sensitive fluorescence dye monochlorobimane (MCB). HaCaT cells (1 × 10^6^ cells/mL) were incubated with 5μM MCB cell tracker for 30min. Intensity of MCB cell tracker fluorescence by GSH was analyzed by the INFINITE M200 Fluoremeter (Tecan Group Ltd., Männedorf, Switzerland) in the fluorescence DAPI region (excitation, 351 nm; emission, 380 nm).

### 3.11. Inflammatory Cytokine Assay

The cells were irradiated with the indicated UVB doses and then incubated with the indicated veratric acid concentration for 12 h. After 12 h, PGE_2_ and IL-6 concentrations in the culture supernatant were measured using ELISA kits (R&D Systems, Minneapolis, MN, USA), according to the manufacturer’s instructions.

### 3.12. Clinical Study of Skin Recovery Effect of Veratric Acid on UV-induced Damage Skin

Eighteen female subjects (average age: 41.8 ± 4.1 years) with Fitzpatrick skin type I, II,III participated in this study. The erythema was induced on the forearms with 1.5MED of individual subjects. The erythema was induced on the forearms with 1.5 MED of individual subjects by Multiport UV Solar Simulator (Solar Light Co., Philadelphia, PA, USA). The two test sites (including non-treatment site) were UV irradiated. At 0 day (after UV irradiation), 1, 2, 3 and 6 days after application of the test product (veratric acid 0.05%), clinical scoring of erythema was assessed by 2 researchers according to a 6-scale scoring method (0~4 grade). The erythema index was evaluated using Mexameter^®^ MX18 (C+K). Erythema index on the test sites (UV-induced damage area on a forearm) were evaluated 3 times before treatment (base line: before UV irradiation), at 0 day (after UV irradiation), 1, 2, 3 and 6 days after application. This study was approved by the ethics committee of the DERMAPRO/Skin Research Center (Seoul, Republic of Korea), and subjects gave written informed consent.

### 3.13. Human Skin Primary Irritation Test

Thirty healthy Korean subjectswith Fitzpatrick skin type I, II,III were selected on the basis of inclusion and exclusion criteria, and written consent was obtained in each case.The average age was 38.1 years (range 24–47: all females). The subjects had no history of allergic contact dermatitis, nor had they used topical or systemic irritant preparations in the previous 1 month. Veratric acid (0.1%) formulated with squalene was prepared and applied. The patches (chambers) stayed in place for 48 h. Once the patches were removed, a reading was done after 48 and the 72 h later, a reading was scored according to a modified criteria proposed by Frosch and Kligman [27] andCosmetic, Toiletry, and Fragrance Association (CTFA) guidelines [28] as follows: 0 = no reaction; 1 = slighterythema, spotty or diffuse; 2 = moderate uniform erythema; 3 = intense erythema with ethema; 4= intense erythema with edema and vesicles. This study was approved by the ethics committee of the DERMAPRO/Skin Research Center, and subjects gave written informed consent.

### 3.14. Statistical Analysis

All data are expressed as mean ± SD. Differences between the control and treatment group were evaluated by Student’s *t-*test using Statview software (Abacus Concepts, Piscataway, NJ, USA). A *p* < 0.01 was considered statistically significant [29].

## 4. Conclusions

In conclusion, the data acquired in this study demonstrate that veratric acid can reduce UV induced skin injuries (DNA damage, redox state inbalance and inflammation), and is safe to use. Veratric acid could be an effective therapeutic agent providing protection against UVB-induced skin disorders.
